# Gastroenteropancreatic neuroendocrine neoplasms: genes, therapies and models

**DOI:** 10.1242/dmm.029595

**Published:** 2018-02-01

**Authors:** Kenta Kawasaki, Masayuki Fujii, Toshiro Sato

**Affiliations:** 1 Department of Gastroenterology, Keio University School of Medicine, Tokyo 160-8582, Japan; 2 Department of Surgical Oncology, The University of Tokyo, Tokyo 113-8654, Japan

**Keywords:** GEP-NENs, Neuroendocrine cancer, Neuroendocrine tumor, Organoids, Rare disease modeling

## Abstract

Gastroenteropancreatic neuroendocrine neoplasms (GEP-NENs) refer to a group of heterogeneous cancers of neuroendocrine cell phenotype that mainly fall into one of two subtypes: gastroenteropancreatic neuroendocrine tumors (GEP-NETs; well differentiated) or gastroenteropancreatic neuroendocrine carcinomas (GEP-NECs; poorly differentiated). Although originally defined as orphan cancers, their steadily increasing incidence highlights the need to better understand their etiology. Accumulating epidemiological and clinical data have shed light on the pathological characteristics of these diseases. However, the relatively low number of patients has hampered conducting large-scale clinical trials and hence the development of novel treatment strategies. To overcome this limitation, tractable disease models that faithfully reflect clinical features of these diseases are needed. In this Review, we summarize the current understanding of the genetics and biology of these diseases based on conventional disease models, such as genetically engineered mouse models (GEMMs) and cell lines, and discuss the phenotypic differences between the models and affected humans. We also highlight the emerging disease models derived from human clinical samples, including patient-derived xenograft models and organoids, which may provide biological and therapeutic insights into GEP-NENs.

## Abstract (precis)

**Summary:** Gastroenteropancreatic neuroendocrine neoplasms have been an understudied entity with limited treatment options. We discuss the disease models, including emerging organoids, that may provide biological and therapeutic insight into these diseases.

## Introduction

Gastroenteropancreatic neuroendocrine neoplasms (GEP-NENs) were originally identified as rare diseases occurring in the gastrointestinal tract and pancreas and displaying distinctive histopathological features from those of conventional gastroenteropancreatic epithelial cancers (Bosman et al., 2010). Unlike the more common gastroenteropancreatic cancers characterized by gland-forming carcinomas, GEP-NENs are characterized by the loss of epithelial tubular gland structures, and by their diffuse expression of neuroendocrine markers (see Box 1 for a glossary of terms). GEP-NENs are broadly classified into two histopathological subtypes: gastroenteropancreatic neuroendocrine tumors (GEP-NETs) and gastroenteropancreatic neuroendocrine carcinomas (GEP-NECs) (Table 1) (Bosman et al., 2010; Lloyd et al., 2017).

**Box 1. Glossary**

**Chronic atrophic gastritis:** chronic inflammation of the gastric mucosa usually associated with a persistent *Helicobacter pylori* infection.

**Complete response (CR):** the state of a patient whose cancer disappears because of the treatment.

**Gastrointestinal adenocarcinoma:** gastrointestinal-derived malignant tumor of differentiated, mucus-secreting glands.

**Hypergastrinemia:** elevated levels of the digestive hormone gastrin, which induces gastric acid secretion (gastrin is released by G cells in the antrum of the stomach).

**Median survival:** the length of time from either the date of diagnosis or the start of treatment for a disease to when half of the patients are still alive.

**Neuroendocrine marker:** an immunoreactive marker indicating the neuroendocrine differentiation of a tissue. Chromogranin A, synaptophysin and CD56 are used as neuroendocrine markers for GEP-NENs. Chromogranin A is a neuroendocrine secretory protein, synaptophysin is a synaptic vesicle glycoprotein present in neuroendocrine cells and CD56 is a neural cell adhesion molecule.

**Refractory:** recurrence of disease after treatment.

**Resectable:** a tumor or lesion that can be treated surgically, often because it is localized.

**Response rate (RR):** percentage of patients whose cancer shrinks or disappears because of treatment.

**Unresectable:** a tumor or lesion that cannot be removed surgically, as in the case of metastatic cancer.

**Table 1.**
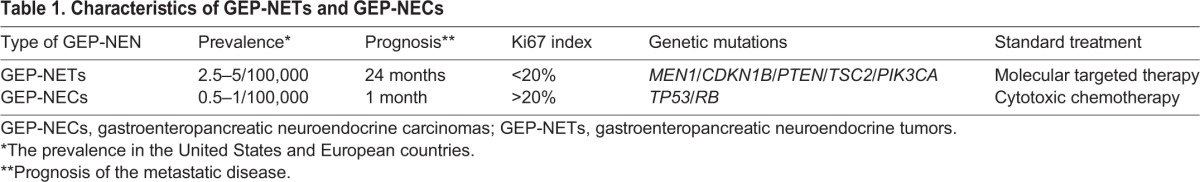
**Characteristics of GEP-NETs and GEP-NECs**

The annual worldwide incidence of GEP-NENs has increased, with a fivefold increase over the past 30 years in the United States – from 1.09 to 5.25 cases per 100,000 persons – possibly due to improvements in endoscopic cancer screening (Yao et al., 2008; Korse et al., 2013). This increase in the incidence of GEP-NENs has resulted in greater attention being paid to these diseases (Yao et al., 2008; Modlin et al., 2003). GEP-NENs originate from various different gastrointestinal tissues, including the stomach, duodenum, jejunum/ileum, pancreas, colon and rectum (Yao et al., 2008). The global incidence of GEP-NENs in each organ is comparable, with 0.3–0.6 cases of GEP-NETs per 100,000 persons and 0.04–0.14 cases of GEP-NECs per 100,000 persons (Yao et al., 2008; Korse et al., 2013).

GEP-NETs are characterized by slow proliferation and initially occur as a localized disease. But treatment delays can result in the tumor's metastatic progression and death (Yao et al., 2008). GEP-NETs are usually asymptomatic until they metastasize, but tumor subtypes producing specific hormones such as insulin (termed insulinoma) and glucagon (termed glucagonoma) often present with hormone-associated symptoms during the localized stages of disease. In contrast, GEP-NECs often progress rapidly and are accompanied by multiple synchronous distant metastases upon diagnosis, leading to a poor prognosis, with patients having only one-sixth of the overall survival rate of those with GEP-NETs (Yao et al., 2008). Radical surgical treatments, including metastasectomy (resection of the metastasis), have been found to improve the prognosis of patients with resectable (Box 1) GEP-NETs, but not that of GEP-NEC patients (Janson et al., 2014). Although several molecular targeted therapies for GEP-NETs and cytotoxic chemotherapies for GEP-NECs have been introduced as standard treatments, the 5-year overall survival of patients with unresectable (Box 1) GEP-NENs has not improved and the treatment options remain limited (Yao et al., 2008).

Disease models are often used to gain new insights into the etiology and biology of human neoplasms and to develop novel treatments. These models include genetically engineered mouse models (GEMMs), cell lines and patient-derived xenograft (PDX) models. However, despite recent efforts, the establishment and application of GEP-NEN disease models have been limited, primarily due to the relatively small number of individuals affected by GEP-NENs. Considering the recent rise in incidence and the poor prognosis of these diseases, it is important to develop GEP-NEN disease models that more accurately reflect the biology of human GEP-NEN tissues in terms of diagnostic criteria and genetic alterations.

In this Review, we provide an overview of the hallmark clinical features, diagnostic criteria, genetic background and currently available models of GEP-NENs. We also highlight the extent to which these models recapitulate human tumor biology and the major insights that have been gleaned from recent studies. We further discuss how emerging organoid models could provide a useful new platform for GEP-NEN disease modeling.

## Diagnostic criteria for GEP-NENs

As mentioned, GEP-NENs are largely divided into GEP-NETs and GEP-NECs, according to the classification criteria defined by the World Health Organization (WHO) (Bosman et al., 2010; Lloyd et al., 2017). A GEP-NENs diagnosis is based on the loss of epithelial tubular gland structures, the diffuse expression of neuroendocrine markers (particularly of chromogranin A, synaptophysin and CD56) and the proliferative cell rate, as represented by the Ki67 index and the mitotic count (Bosman et al., 2010; Lloyd et al., 2017). Ki67 is a marker of proliferating cells and, through this index, GEP-NENs are sub-classified into three groups according to the 2010 WHO classification (Bosman et al., 2010): NET G1, NET G2 and NEC. NET G1 cells are well-differentiated and have a Ki67 index of <2 and a mitotic count of <2 per 10 high-power fields (HPF). This indicates that less than 2 proliferative cells are observed in 10 HPF. NET G2 cells have a Ki67 index of 3–20% and a mitotic count of 2–20 per 10 HPF. Poorly differentiated GEP-NECs are defined as NEC. These cells have a Ki67 index of >20% and a mitotic count of >20 per 10 HPF. Recently, a patient population that does not fit into the classification of typical GEP-NECs was reported (Sorbye et al., 2013) and, accordingly, the WHO classification of pancreatic NENs has been updated. In this revision, pancreatic NECs in the former classification have been divided into two disease subsets on the basis of the Ki67 index and histological morphology. These subsets are called pancreatic NET G3 and pancreatic NEC G3 (Lloyd et al., 2017). Pancreatic NET G3 has a Ki67 index ranging from 20 to 55% with well-differentiated morphology, and patients have a significantly longer survival time compared to individuals affected by typical pancreatic NECs (Sorbye et al., 2013). Pancreatic NEC G3 has a Ki67 index >55% with poorly-differentiated morphology, and is characterized by a poor prognosis.

The use of the Ki67 index as a disease marker, however, requires caution since it might not reflect the biology and the heterogeneity of the disease. The Ki67 index is highly influenced by the surrounding tumor microenvironment and by therapeutic interventions. Thus, it might reflect these biasing factors rather than the intrinsic properties of the tumor cells (Sorbye et al., 2013; Singh et al., 2014). Even in the same patient, metastatic tumors tend to have a higher Ki67 index than do primary tumor cells (Hentic et al., 2011; Delektorskaya et al., 2013). To overcome these limitations of the Ki67 index, genetic abnormalities of GEP-NENs have been introduced into the revised WHO classification, which we discuss in the next section (Lloyd et al., 2017). Currently, these classification changes are limited to pancreatic NENs; their application to gastrointestinal NENs remains to be established.

## Genetics of GEP-NENs

Revealing the mutational landscape of GEP-NENs is important for deepening our understanding of the biology of these diseases. Among recent insights, several mutations in cell-cycle-related genes have been discovered in GEP-NENs through advances in targeted gene sequencing.

Genetically, GEP-NETs can be categorized as being familial in origin (where individuals inherit a predisposing mutation) or sporadic (where an inherited susceptibility allele is not found). To date, at least four familial GEP-NET syndromes have been reported: multiple endocrine neoplasia type 1 (MEN1), tuberous sclerosis complex (TSC), Von Hippel–Lindau (VHL) and neurofibromatosis type 1 (NF1) (Crona and Skogseid, 2016). MEN1 is an autosomal dominant syndrome, the most common among the four, and causes pancreatic NETs through an inactivating mutation in *MEN1*, which encodes menin (Jiao et al., 2011)*.* The function of menin is unknown, but the loss of this protein causes cell cycle progression through the downregulation of CDKN2C and CDKN1B, which both negatively regulate the cell cycle (Karnik et al., 2005). TSC is caused by mutations in *TSC1* and *TSC2*, which encode the proteins tuberous sclerosis complex 1 and 2, respectively. These mutations cause defects in the mammalian target of rapamycin (mTOR)–AKT pathway, which also regulates the cell cycle (Schmelzle and Hall, 2000). VHL is caused by loss-of-function mutations in the *VHL* tumor suppressor gene. And, NF1 is caused by loss-of-function mutations in *NF1*, which encodes neurofibromin, a protein that functions as a tumor suppressor through repressing the RAS pathway (Rad and Tee, 2016). Recently, two novel familial NETs have been reported. One is characterized by loss-of-function mutations in *ATP4A*, which encodes a proton pump (Calvete et al., 2015), and the other is characterized by loss-of-function mutations in *IPMK*, which encodes a member of the inositol phosphokinase family involved in regulation of p53-mediated apoptosis (Sei et al., 2015).

The genetic mutations found in sporadic GEP-NETs also alter cell cycle regulatory pathways, with the most common mutations occurring in *MEN1* (Scarpa et al., 2017). Recent genetic mutation analyses have also uncovered recurrent mutations in *CDKN1B*, which encodes a cyclin-dependent kinase inhibitor, and in genes that negatively regulate the mTOR–AKT pathway, including in *DEPDC5* (encoding a suppressor of the mTOR–AKT pathway, GATOR1 complex), *PTEN* (encoding a tumor suppressor, phosphatidylinositol-3,4,5-trisphosphate 3-phosphatase), *TSC1* and *TSC2* (Jiao et al., 2011; Banck et al., 2013; Francis et al., 2013; Scarpa et al., 2017). Mutations in cell cycle regulatory pathway genes are also found in GEP-NECs; specifically, recurrent mutations in *TP53*, which encodes p53, a transcription factor and regulator of apoptosis (Shaw et al., 1992), and in *RB* (retinoblastoma), which encodes a key tumor suppressor that regulates the cell cycle (Whyte et al., 1988). In contrast to GEP-NETs, for which only 3% of tumor samples present with *TP53* mutations, GEP-NECs harbor *TP53* mutations in 90–95% of cases (Furlan et al., 2005; Yachida et al., 2012). Additionally, *RB* mutations occur in 60–75% of all GEP-NECs (Pizzi et al., 2003).

These data suggest that cell cycle dysfunction is the fundamental cause of GEP-NENs, and that the differences in the clinical features and malignancy of these neoplasms arise from their different pathway mutations; mTOR pathway mutations in GEP-NETs, and *TP53* and *RB* pathway mutations in GEP-NECs. The high incidence of *TP53* and *RB* mutations in GEP-NECs could provide a more accurate means of differentiating GEP-NETs from GEP-NECs, as reflected in the recently revised WHO classification for pancreatic NENs (Lloyd et al., 2017). In this new classification, pancreatic NET G3s have a high Ki67 index (which is indicative of a pancreatic NEC) but do not have *TP53* or *RB* mutations. Further biological investigations are warranted to clarify how other genetic mutations distinguish GEP-NETs and GEP-NECs by affecting their distinct clinical behavior, in order to optimize the diagnostic and treatment strategies for these diseases.

## Current treatment strategies for GEP-NENs

Treatment strategies for GEP-NENs are largely divided according to whether tumors are resectable or unresectable. For resectable disease, the main treatment strategy is surgery. For unresectable disease, prognosis and treatment strategies will depend on whether the disease is classified as being GEP-NETs or GEP-NECs, based on the Ki67 index.

### Treating GEP-NETs

The systemic administration of several different drugs, such as streptozocin, somatostatin analogs (such as octreotide and lanreotide), peptide receptor radionuclide therapy (PRRT), everolimus and sunitinib, has been reported to improve the survival of patients with unresectable GEP-NETs (Moertel et al., 1980; Rinke et al., 2009; Caplin et al., 2014; Strosberg et al., 2017; Yao et al., 2011, 2016; Raymond et al., 2011). Streptozocin is a classic cytotoxic agent that shows islet-cell-specific toxicity in rodent models (Bennett and Pegg, 1981). It is selectively transported into pancreatic islet cells via GLUT2 transporters and it has been used since the 1980s to treat islet-cell tumors (such as GEP-NETs, according to the WHO classification) (Moertel et al., 1980). Somatostatin analogs mimic endogenous somatostatin and bind with high affinity to somatostatin receptor 2, which mediates anti-proliferative signals via the MAPK/extracellular signal-regulated kinase (ERK) pathway; as such, somatostatin analogs have been used to control and mitigate symptoms associated with GEP-NETs (Susini and Buscail, 2006). In a further development, somatostatin analogs have been radiolabeled with lutetium-177 (^177^Lu–Dotatate) – a treatment strategy called PRRT (Strosberg et al., 2017). This targeted radiotherapy delivers radionuclides to all tumor cells expressing somatostatin receptors. Everolimus is an mTOR inhibitor targeting mTOR–AKT pathway signals, which are constitutively active as a result of the genetic mutations previously discussed (Scarpa et al., 2017). Sunitinib is a multi-target receptor tyrosine kinase inhibitor. Its targets include the platelet-derived growth factor receptor (PDGFR) and the vascular endothelial growth factor receptor (VEGFR), which both contribute to tumor cell proliferation via angiogenesis (Couvelard et al., 2005).

The response rates (RRs; Box 1) for these treatments are below 30%, highlighting the considerable need for the development of biomarkers for these tumors to achieve improved drug response (Rindi and Wiedenmann, 2012).

### Treating GEP-NECs

The setting up of clinical trials to improve the therapeutic strategies available for patients with GEP-NECs is hampered by the low number of patients with this disease. Nevertheless, GEP-NECs have been treated using the treatment guidelines developed for small-cell lung cancer based on the histological similarities between these cancers. These strategies include using a combination chemotherapy regimen consisting of cisplatin or carboplatin with etoposide or irinotecan (Strosberg et al., 2010). In several studies, this combination has shown a good RR of 40–60%. But, eventually, most cases become refractory (Box 1), and the median survival (Box 1) of these GEP-NEC patients is only 15–19 months (Moertel et al., 1991; Mitry et al., 1999). Furthermore, no evidence-based second-line chemotherapy has been established, and the reported RR of second-line therapies, such as topotecan, temozolomide, paclitaxel, docetaxel, vinorelbine and gemcitabine, are low at approximately 0–20% (Strosberg et al., 2010; Janson et al., 2014; Walenkamp et al., 2009; Welin et al., 2011). GEP-NECs are more radiosensitive than are GEP-NETs due to their vigorous proliferation (Casas et al., 1997), but the efficacy of the radiation therapy is low, with little potential for a complete response (CR; Box 1) due to refractory disease. The development of novel treatment strategies, through the identification of suitable biomarkers that have been validated in preclinical disease models, is required to achieve better patient outcomes. The current preclinical disease models are discussed in the next section.

## Current disease models of GEP-NENs

Establishing experimental models that recapitulate GEP-NENs is important to increase our understanding of the biology of these diseases and to develop novel targeted therapies to treat them. Several disease models of GEP-NENs have been reported, and have contributed to drug development of the current standard of care. However, these disease models are limited by their insufficiency to recapitulate the genetic mutations and diagnostic features of the human neoplasms. An appropriate preclinical disease model would be one that mimics the genetic alterations that feature in GEP-NENs and fulfills the diagnostic criteria that can be applied for GEP-NEN drug screening and development. Here, we provide an overview of the current disease models for GEP-NENs, including emerging organoid models, and highlight their advantages and limitations, and some of the insights that have been gained from their characterization (Table 2).

**Table 2.**
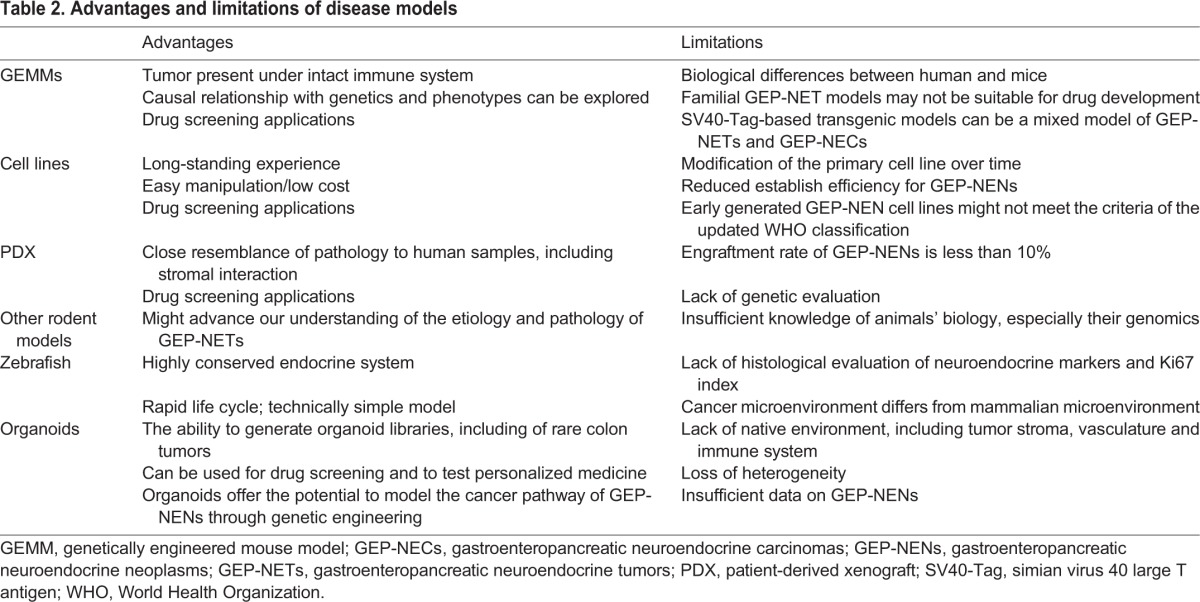
**Advantages and limitations of disease models**

### Genetically engineered mouse models

GEMMs are a key tool in cancer research as they enable tumors to be evaluated in the presence of an intact immune system, and allow the causal relationship between genetic mutations and phenotypes to be explored. GEMMs can also be used in a variety of ways, such as for drug target validation and for testing novel therapies, for biomarker discovery, and for evaluating treatment toxicity (Sharpless and Depinho, 2006).

GEMMs for GEP-NETs include genetic knockout models and simian virus 40 (SV40)-large T antigen (Tag)-based transgenic models. Genetic knockout models have been generated by engineering germline mutations in mouse strains (Table 3). Whereas the homozygous knockout of *Men1* causes embryonic lethality at E11.5–E12.5, *Men1* heterozygous knockout mice develop tumors in multiple organs and develop certain features found in patients with MEN1 (Crabtree et al., 2001). GEMMs that carry an *Atp4a* mutation that has been found in patients with familial gastric NETs recapitulate hypergastrinemia (Box 1) and develop gastric NETs (Calvete et al., 2016; Syder et al., 2004). The tumors in *Men1* and *Atp4a* knockout mouse models display a Ki67 index of less than 20% and fulfill the histopathological criteria for GEP-NETs, underscoring their suitability for modeling GEP-NETs (Shen et al., 2009; Calvete et al., 2016). But these genetic knockout models have been mainly characterized and validated to study the causal relationship between genetic mutations and phenotypes rather than to screen novel therapeutic options, based on their characteristics as hereditary diseases.

**Table 3.**
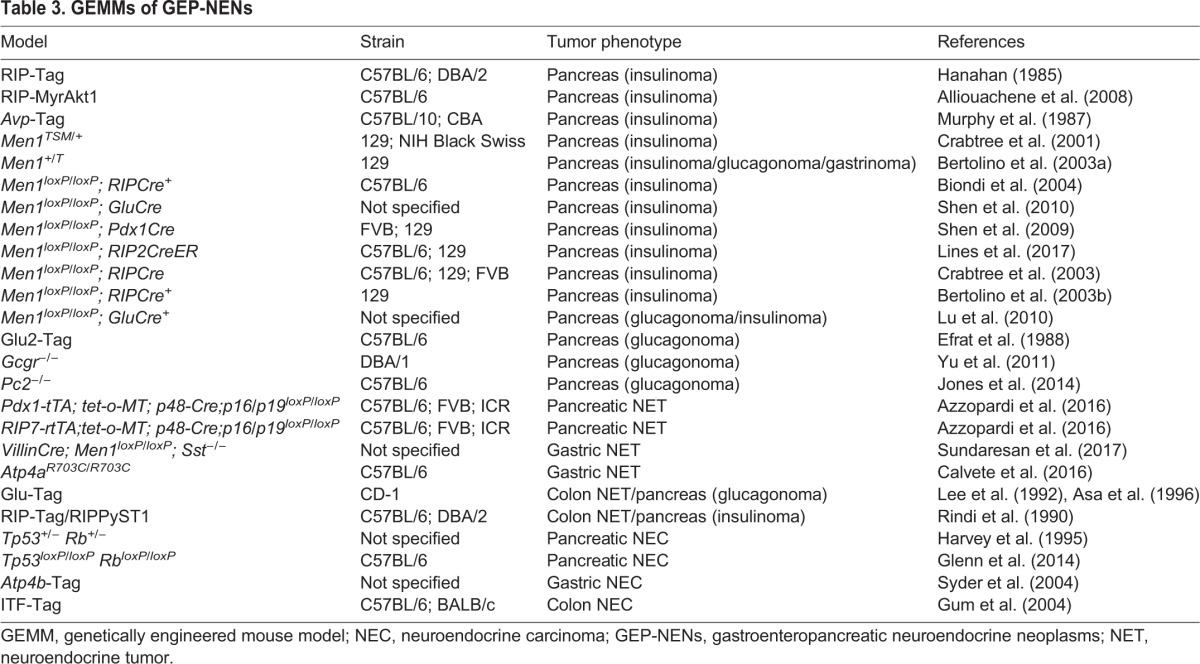
**GEMMs of GEP-NENs**

The rat insulin promoter (RIP)-Tag mouse model is a representative model for SV40-Tag-based transgenic models, in which the RIP drives the expression of Tag specifically in islet cells, and β-cells become highly proliferative in response to SV40-Tag expression (Hanahan, 1985). The RIP-Tag mouse has been widely used as a model for GEP-NETs (particularly of the insulinoma tumor subtype) and has been used to evaluate the therapeutic potential of everolimus and sunitinib (Casanovas et al., 2005). The promising preclinical results obtained were subsequently validated in human clinical trials (Yao et al., 2016; Raymond et al., 2011). Although Tag-based transgenic models have also been generated to recapitulate other GEP-NETs, such as glucagonoma (in the preproglucagon promoter-driven Tag mouse) and colonic NET (using the same Tag model strategy) (Efrat et al., 1988; Asa et al., 1996), they have not been used as much as the RIP-Tag model to evaluate drug responses (Table 3).

GEMMs for GEP-NECs are scarce, probably due to the lack of biological understanding of the disease; additionally, efforts have focused on modeling neuroendocrine cancers in other organs, such as the models for small-cell lung cancer and prostate neuroendocrine cancer. Comprehensive genomic sequencing of small-cell lung cancer has identified recurrent loss-of-function mutations in *TP53* and *RB1* (George et al., 2015)*.* GEMMs harboring these same mutations recapitulate the histological traits of small-cell lung cancer (Meuwissen et al., 2003). Genetic mutations in *TP53* and *RB1* have also been found in prostate neuroendocrine cancer. Recently, the development of murine prostate neuroendocrine cancer that closely resembles human prostate neuroendocrine cancer was reported in *TP53* and *RB1* knockout GEMMs (Ku et al., 2017; Mu et al., 2017). This model has allowed the study of how therapeutic resistance develops in prostate cancer through lineage plasticity with *TP53* and *RB1* mutations, and overexpression of *SOX2* and *EZH2*. Just as in human patients undergoing androgen therapy, these mutations reproduced the phenotypic changes observed in sequential periods where androgen-receptor-dependent luminal cells seem to turn into androgen-receptor-independent basal cells. This phenomenon might reflect an adaptive response to treatment, through biological selective pressure. Although this might be different in the development of GEP-NECs, this observation gives us an important insight into the progression of the disease (Rickman et al., 2017). Interestingly, Parisi et al. have reported that *TP53* and *RB1* knockout mice develop not only prostate and female reproductive tract neuroendocrine cancers, but also conventional colon adenocarcinoma instead of colon NECs (Parisi et al., 2015). Other knockout mouse models of *TP53* and *RB1* have not been evaluated as disease models of GEP-NECs with respect to their expression of neuroendocrine markers and Ki67 index; as such, the extent to which these models resemble these human neoplasms remains unknown (Harvey et al., 1995; Glenn et al., 2014). Although difficult to conclude given the limited number of reports, it remains possible that additional factors, other than the *TP53* and *RB1* mutations, might be needed to recapitulate the features of GEP-NECs.

Aside from the obvious biological differences between mice and humans, GEMMs present other limitations, such as the latency in tumor development and growth. In addition, most GEP-NETs are not familial, and it remains to be confirmed whether the findings obtained from GEMMs that harbor germline mutations can be generalized to sporadic GEP-NETs. For the SV40-Tag-based transgenic model, it should also be noted that this model might not reconstitute the precise biology of GEP-NETs. Although the validity of the RIP-Tag mouse model has been supported by clinical response, a recent report has revealed that a fraction of pancreatic tumors in this mouse model exhibit a Ki67 index of over 80%, which suggests that tumors in this model are heterogeneous and consist of both GEP-NETs and GEP-NECs (Hunter et al., 2013). A mixture of GEP-NETs and GEP-NECs has not as yet been reported in human tumor samples, suggesting that this neoplasm mixture is unique to this mouse model. The RIP-Tag mouse model was validated as a model of insulinoma on the basis of insulin expression in the tumor. Thus, it remains unknown whether this GEMM recapitulates the genetic background of human GEP-NETs and their diagnostic histological traits, such as the Ki67 index. Additionally, Tag promotes the inactivation of the target genes, such as *Tp53* and *Rb1*, by forming a specific complex in a non-physiological manner (Linzer and Levine, 1979; DeCaprio et al., 1988); thus, it may be modeling GEP-NECs instead of GEP-NETs (Chandrasekaran et al., 1996; Modlin et al., 2008). Indeed, it has been suggested that SV40-Tag expression under the control of the intestinal trefoil factor (ITF) promoter could be used to generate a mouse model of colon cancer that closely resembles small cell carcinoma, although the Ki67 index of this model remains to be evaluated (Gum et al., 2004). These examples highlight the need for GEMMs to be carefully evaluated, both genetically and histologically, before they are used to model a particular GEP-NEN.

### Cell-based models of GEP-NENs

Cell lines are frequently used to model cancer, and have made a considerable contribution to cancer research over recent decades. The advantages of using cell lines as disease models include their ease of manipulation and low cost, which also facilitates their use in drug screening.

To date, several different GEP-NEN cell lines have been established, including nine GEP-NET and six GEP-NEC human cell lines, and six rodent GEP-NEN cell lines (Table 4). The human GEP-NET cell lines, BON1 and GOT1, develop into tumors with neuroendocrine characteristics when introduced as xenografts into immunocompromised mice (Evers et al., 1991). The genome of the BON1 cell line has been sequenced to reveal that these cells carry mutations in *TP53*, *TSC2* and *NRAS* (Vandamme et al., 2015; Boora et al., 2015). These cell lines have also been used to develop drugs to treat GEP-NETs. BON1 has been used to test somatostatin analogs and everolimus (Zitzmann et al., 2007; Kidd et al., 2007). The KRJ-I cell line, derived from a patient's small-intestinal NET, has been used to test somatostatin analogs (Evers et al., 1991; Ishizuka et al., 1992). The cell line GOT1 has also been used successfully to test ^177^Lu–Dotatate (Nilsson et al., 2004). The responses of these cell lines to these drugs have been evaluated based on cell growth inhibition and apoptosis induction, and the results have been consistent with the clinical responses (tumor shrinkage) seen in GEP-NET patients. With further assessment of their histology, drug responses and genetic mutations, these cell lines could be developed as appropriate GEP-NET models.

**Table 4.**
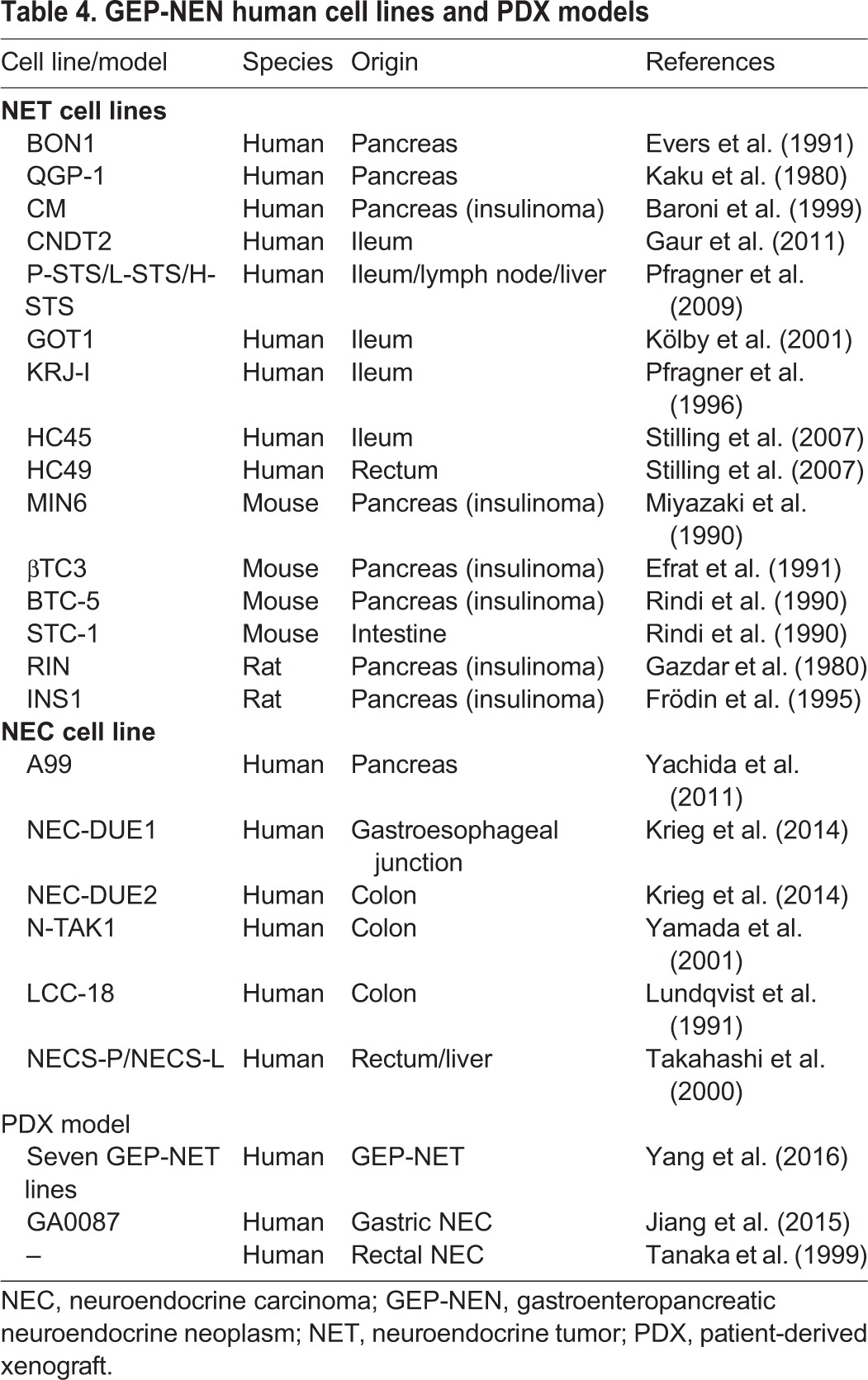
**GEP-NEN human cell lines and PDX models**

Recently, a new method for culturing primary pancreatic NET cells using bovine extracellular matrix has been reported (Mohamed et al., 2014, 2017) and used to establish 30 pancreatic NET lines. This culturing method can maintain cells that have neuroendocrine characteristics, allowing for the assessment of their genomic mutations and drug responses (to somatostatin analogs and everolimus) (Mohamed et al., 2014, 2017). One of these pancreatic lines has a high Ki67 index of 90% and should therefore be considered a pancreatic NEC line. In addition, 16 pancreatic NET primary cell culture lines, including two pancreatic NEC lines that respond to everolimus, have been established by another laboratory (Falletta et al., 2016). The creation of these pancreatic NEN cell lines provides new preclinical models for researchers to use and are therefore expected to contribute significantly to advancing research into GEP-NENs. The three GEP-NEC cell lines (NEC-DUE1, NEC-DUE2 and A99) have been evaluated for their expression of neuroendocrine markers and for their high Ki67 index using xenograft models (Yachida et al., 2011; Krieg et al., 2014). These NEC cell lines fulfill the current classification of GEP-NECs and can be considered as appropriate models to use. Although the response of NEC-DUE1 and NEC-DUE2 to existing drugs has been evaluated (Krieg et al., 2014), these NEC cell lines have yet to contribute to new drug development.

Cell lines carry with them intrinsic limitations, such as the occurrence of genetic changes over time in culture, or their reduced establishment efficiency, but, especially for GEP-NEN cell lines, the updated WHO classification may mean they need to be re-cataloged (for example as NET G3). CNDT2 is a cell line derived from a liver metastasis of a patient with a primary ileal NET. When initially transplanted into mice, this cell line developed as a tumor resembling the patient's tumor histology (Van Buren et al., 2007). However, a subsequent study reported that the tumors formed by transplanted CNDT2 cells lacked secretory granules and neuroendocrine characteristics, such as chromogranin A staining – a hallmark of GEP-NETs – and that the tumor cells had gene expression patterns that were different from those of GEP-NETs (Van Buren et al., 2007; Ellis et al., 2010). Disease heterogeneity or the occurrence of genetic changes over time in culture might explain these results, highlighting a need for caution when using any GEP-NEN cell line. The efficiency with which cell lines from GEP-NENs can be established is presumably low, and may indicate the artificial selection of the few tumor cells that can adapt to standard culture conditions, highlighting the need for caution when interpreting results from current GEP-NEN cell line studies. However, the low establishment efficiency may also be due to small amounts of available starting material and low mitotic rates (Rockwell, 1980; Modlin et al., 2005; Kasai et al., 2016). In addition, cell lines validated as models of GEP-NENs according to the past WHO classifications for these diseases may no longer meet the criteria of the updated WHO guidelines, requiring extra caution and biological confirmation of the cell lines before their use.

### Patient-derived xenograft models

PDX models offer an alternative research tool to cell models of GEP-NENs. PDX models are made by transplanting primary human tumor cells into an animal. These models have the advantage of retaining the histological and genetic characteristics of the primary tumor and provide valuable disease-modeling platforms that are amenable to various *in vivo* applications, such as drug screening (including high-throughput screening), based on their relevance to patients' tumors, including stromal conservation (Peng et al., 2013; Gao et al., 2015). Recently, an approach called co-clinical trials has been proposed that enables a new agent to be studied in parallel in human patients and in a PDX model derived from a patient's primary tumor. This approach offers a personalized medical approach to treat these tumors (Hidalgo et al., 2014). Seven GEP-NET PDX models and two GEP-NEC PDX models that form tumors resembling the original patient's tumors have been successfully established in mice (Table 4) (Tanaka et al., 1999; Jiang et al., 2015; Yang et al., 2016). One gastric NEC model, GA0087, evaluated for its drug responses has shown extended survival relative to untreated controls in response to treatment with cisplatin, which is concordant with clinical results (Jiang et al., 2015). Thus, GEP-NEN PDX models can also be considered to be appropriate models to use for drug screening and biological evaluation. However, GEP-NENs have an engraftment success rate of less than 10% (Yang et al., 2016). Some of the PDX models of GEP-NETs exhibit a Ki67 index of 70–90%, and thus more closely match GEP-NECs under the current diagnostic criteria (Yang et al., 2016). In addition, there has been insufficient genetic evaluation of PDX models of GEP-NETs, although their histology resembles that of human tumors. The recent evaluation of copy number alterations in PDX models revealed the selection of minor clones, causing discrepancies with patient tumors (Ben-David et al., 2017). Thus, even for established GEP-NET PDX models, careful evaluation is required before they are used.

### Other rodent models of GEP-NENs

Animal models in which GEP-NETs have developed incidentally or spontaneously are also used as tools for investigating these tumors. One example of such a model is a transgenic rat that was originally developed as a model of hepatocarcinogenesis and unexpectedly developed pancreatic NETs (Haas et al., 1999, 2000). This rat expressed SV40 under the control of a phosphoenolpyruvate carboxikinase (PEPCK) promoter (Haas et al., 1999). This model remains to be histologically evaluated for its Ki67 index and for neuroendocrine marker expression. Furthermore, the mechanism of NET formation in the rat remains unknown, and they suffer from the same limitations as the RIP-Tag mouse model, due to the expression of SV40.

The Natal multimammate mouse (*Mastomys natalensis*) is an African rodent that tends to develop multi-centric gastric NETs as it ages (Snell and Stewart, 1969). It has been used as an experimental model of chronic atrophic gastritis (Box 1), since the development of gastric NETs in it can be enhanced by inducing hypergastrinemia using histamine receptor blockers and proton pump inhibitors (Nilsson et al., 1992). Hypergastrinemia has also been studied in spontaneous hypergastrinemic cotton rats (*Sigmodon hispidus*) and in a Mongolian gerbil (*Meriones unguiculatus*) model of gastric NETs induced by high doses of proton pump inhibitors (Fossmark et al., 2004; Tsukamoto et al., 2011). These models are good candidates to use for investigating the development and treatment of GEP-NETs; however, their use is limited by our poor understanding of these species, including their genomics. Extensive efforts for the development of disease models have led to non-rodent animal models – zebrafish – to be introduced.

### Zebrafish models of GEP-NENs

The zebrafish (*Danio rerio*) has been used to study genetics and development for over three decades (Streisinger et al., 1981). Given its highly conserved endocrine system, similar to that of humans (Vitale et al., 2014), it is now a new research tool for studying neuroendocrine neoplasms. The advantages of this model include its short generation cycle, high reproductivity and genetic tractability. A transgenic zebrafish model of a pancreatic NEC has been generated by targeted expression of the human *MYCN* oncogene in pancreatic islet cells under the control of the z-myod or core-zmyod promoter (Yang et al., 2004). Additionally, two GEP-NET xenotransplantation models have also been generated using cell lines developed from primary human tumor samples (Gaudenzi et al., 2017). However, these GEP-NEN models need to undergo an in-depth evaluation, including a histological analysis to measure the Ki67 index of tumors and an assessment of neuroendocrine marker expression. In addition, zebrafish models of GEP-NENs cannot recapitulate the tissue niche or cancer microenvironment associated with human tumors (Zhang et al., 2015). The zebrafish models have their limitations, but should be regarded as another tool that may help us grow our biological understanding of GEP-NENs.

### Modeling GEP-NENs in organoids

An organoid is a three-dimensional tissue grown in culture that resembles a particular organ in its morphology and cell lineage characterization. Organoids are generated *in vitro*, often from adult stem cells, using a basement membrane matrix (such as Matrigel) for cells to adhere to, and various growth factors to stimulate differentiation (Sato et al., 2009). The advent of the organoid culture system, which artificially reproduces stem cell niche environments in a dish, has facilitated the *in vitro* expansion of primary epithelial cells derived from a variety of tissues (Date and Sato, 2015). Although each tissue depends on a unique combination of niche factors for organoid culture, most organoids require three essential niche factors: Wnt/R-spondin-1, the BMP inhibitor Noggin and epidermal growth factor (EGF) (Sato et al., 2009). Two additional niche factors, an activin receptor kinase inhibitor and a p38 MAP kinase inhibitor, enable human colon epithelial cells to develop as organoids in culture (Sato et al., 2011). Organoids can be passaged for years while retaining genetic and phenotypic stability (Blokzijl et al., 2016). Importantly, the organoid culture technique can be used to create organoids from diseased epithelia obtained from patients.

**Fig. 1.**
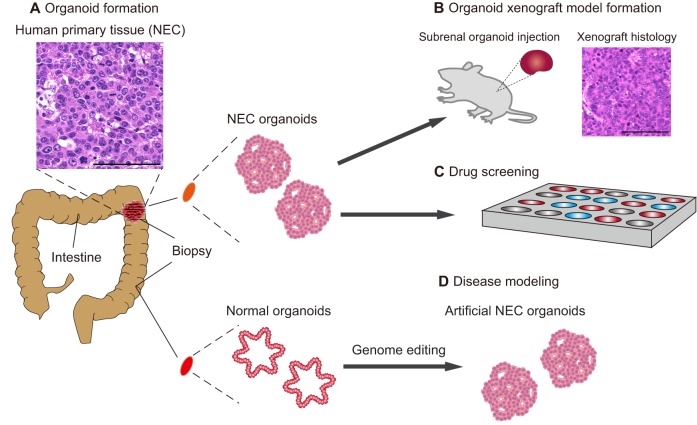
**Disease modeling of GEP-NENs using organoids.** (A) Organoids are constructed in culture medium, and patient-specific organoids can be derived from a patient's primary tissue (shown in the inset; scale bar: 100 µm). This schematic shows biopsies being taken from a human intestine, the upper biopsy contains neuroendocrine carcinoma (NEC) and the lower biopsy contains normal intestinal tissue. Organoids are derived from both biopsies to generate NEC organoids and normal intestinal organoids. (B) The NEC organoids can be transplanted into rodent models to create xenograft models. The histology of a xenograft model, reconstituting the patient's histology as determined by H&E staining, is shown. Scale bar: 100 µm. (C) Organoids can also be used for drug screening, and (D) disease modeling. In disease modeling, artificial NEC organoids can be constructed by using genome editing to introduce specific genetic mutations into normal colon organoids.

Recently, two research groups (including our own) have developed a colon cancer organoid biobank, which contains a diverse range of human colorectal tumor subtypes (van de Wetering et al., 2015; Fujii et al., 2016). We generated a library called the colorectal tumor organoid library (CTOL), which includes two colorectal NECs (which have a Ki67 index of 60 and 100%) (Fujii et al., 2016). Upon xenografting to immunocompromised mice, these organoids formed tumors that histologically resembled the colorectal NECs of the original patient, including synaptophysin and chromogranin expression. But, given our lack of knowledge about the common gene expression signature of colorectal NEC tissues, the extent to which the profile in our NEC organoids resembles that of primary colorectal NECs remains to be determined. Nonetheless, it is worth noting that the gene signatures of NEC organoids were distinct from other colorectal tumors, with endocrine markers, such as chromogranin and synaptophysin, being highly expressed (Fujii et al., 2016). *TP53* mutations were found in both NEC organoids, and mutations in several genes associated with colorectal adenocarcinoma, such as *APC*, *BRAF* and *KRAS*, were also found in one of the two NEC organoids (Fujii et al., 2016). There is hope that, once several different lines of GEP-NEN organoids are established, they can be used to conduct ‘*in vitro* clinical trials’ with compounds that have been approved for use in the treatment of other types of cancer. Indeed, high-throughput drug screening systems have already been developed for organoid cultures for other types of cancers, including sporadic colorectal cancer (van de Wetering et al., 2015; Schütte et al., 2017; Pauli et al., 2017) (Fig. 1).

**Fig. 2.**
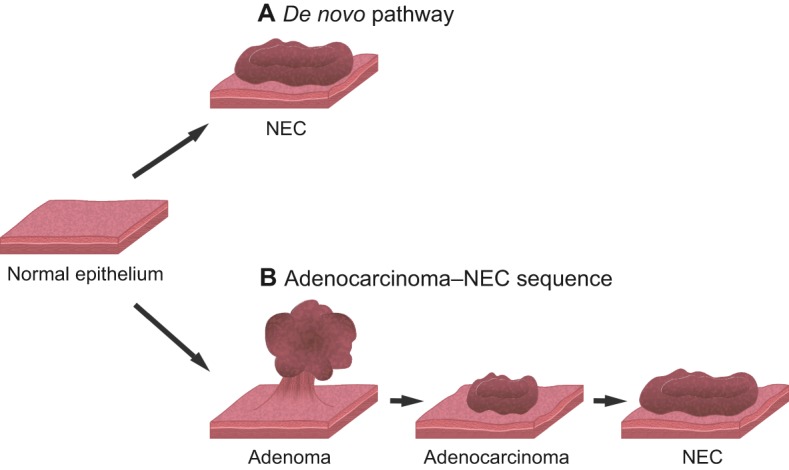
**Hypothetical model of GEP-NECs.** Gastroenteropancreatic neuroendocrine carcinomas (GEP-NECs) are considered to arise from two possible pathways. (A) A *de novo* pathway, in which GEP-NECs form directly from normal intestinal tissue and (B) from adenocarcinomas via several steps. This latter pathway stems from the multistep oncogenesis model of conventional colon cancer, which suggests a progression from normal colon epithelium to adenocarcinoma via adenoma. The adenocarcinoma–NEC sequence is currently regarded as the dominant route.

Organoid models of cancer can also be useful for understanding how genetic alterations affect tumorigenesis. In particular, organoid technology has the potential to reveal the biological origin of GEP-NENs, including the involvement of underlying cancer stem cell populations (Rindi and Wiedenmann, 2012). Two tumorigenic pathways have been proposed to explain the etiology of GEP-NECs, of which the progression from conventional adenocarcinoma (adenocarcinoma–NEC sequence) is currently regarded as the dominant route over the *de novo* pathway (Fig. 2) (Vortmeyer et al., 1997; Kawasaki et al., 2017). This theory stems from the oncogenesis model of conventional gastrointestinal adenocarcinomas (Box 1), represented by a multistep oncogenesis process, such as the adenoma–carcinoma sequence in colorectal cancer. Clinically, some colorectal NECs coexists with adenoma or adenocarcinoma, and tumors comprising adenocarcinomas and gastrointestinal NECs are defined as mixed adeno-neuroendocrine carcinomas (MANECs) in the WHO classification (Bosman et al., 2010). Gastrointestinal NECs and conventional gastrointestinal adenocarcinomas share similar genetic mutation patterns, supporting the notion that colorectal NECs originate from adenocarcinomas (Fujii et al., 2016; Kim et al., 2002; Scardoni et al., 2014). Conversely, the co-existence of colorectal NECs and colorectal NETs has not been reported, and, given the commonality of the genetic characteristics between colorectal NECs and adenocarcinomas, well-differentiated colorectal NETs might be less likely to progress into poorly differentiated colorectal NECs (Helpap and Kollermann, 2001; Rickman et al., 2017; Takizawa et al., 2015). However, this theory was derived from retrospective observations, and prospective validation is warranted to further understand the molecular road map of GEP-NENs to improve prognosis and treatment outcomes. The combination of organoid technology and genome editing technology might help to elucidate the molecular etiology of these diseases. The feasibility of this approach has recently been demonstrated by the sequential introduction of mutations in tumor suppressor genes and oncogenes into normal colon organoids, providing a proof of principle for the genetic reconstruction of human colorectal tumorigenesis from normal colon epithelium *ex vivo* (Matano et al., 2015; Drost et al., 2015). The generation of GEP-NETs and GEP-NECs using this approach could provide a definitive clue as to the cell of origin of GEP-NENs and shed new light on the interrelationship between GEP-NETs and GEP-NECs.

Although organoids open up new avenues for GEP-NEN research, their limitations should also be noted. First, organoids lack a native microenvironment that includes the tumor stroma, the vasculature and the immune system. Thus, organoids may not be suitable for evaluating tumor cell interactions with these non-epithelial elements in the native cancer environment. Second, as organoids are generally derived from limited amounts of biopsy or surgical samples, they are oligoclonal and might not represent the heterogeneous nature of a cancer. Third, only two GEP-NEN organoid lines (both from colorectal NECs) have been derived to date, and the lack of GEP-NET organoids renders the current culture system insufficient for modeling GEP-NENs.

## Conclusion

GEP-NENs are heterogeneous diseases that have been understudied for several decades due to the small number of patients who develop these diseases. However, their increasing incidence, poor prognosis and limited treatment options has focused attention on these diseases, stimulating research in this field. Technological advances in clinical diagnosis and genetic analysis have furthered our understanding of the biology of these diseases in recent years. Data suggest that cell cycle dysfunction underlies the etiology of GEP-NENs and that different tumor subtypes feature different pathway mutations: mTOR pathway mutations in GEP-NETs, and *TP53* and *RB* pathway mutations in GEP-NECs. These mutational differences might cause the different clinical features of these tumor subtypes and might influence the resulting treatment options, such as molecular targeted therapy for GEP-NETs and cytotoxic treatment for GEP-NECs. The establishment of these current standard therapies has arisen from studies conducted in GEP-NEN animal models, which were developed through extensive efforts. However, due to the small number of individuals affected by GEP-NENs, and the low success rates in the development of the disease models, the number of animal models remains limited. Additionally, some of the current animal models were validated under the previous WHO classification for GEP-NENs, when the role of genetic mutations was unknown and the terminology for GEP-NETs and GEP-NECs was less clear. Thus, very few of the existing GEP-NEN models have been confirmed clinically and genetically as being informative and accurate. Additional models of GEP-NENs are thus needed to advance this field and to gain new biological insights into the cellular and molecular etiology of these diseases. Modeling GEP-NENs in organoids could provide a new platform for this field and could also enable the reconstruction of the oncogenic steps that lead to GEP-NEN development through genetic engineering. Organoid models have the potential to contribute to the ongoing development of novel treatments and to the identification of the GEP-NEN stem cell of origin, which could lead to the eventual eradication of these diseases. Disease models that faithfully recapitulate GEP-NENs are indispensable for the translation of animal-model-based research to the clinic, to help overcome the limited treatment strategies for these diseases.
